# Five flaps or four? Z-plasty for the release of first webspace burn contractures of the hand

**DOI:** 10.1093/jscr/rjae702

**Published:** 2024-11-18

**Authors:** Alan D Rogers, Syena Moltaji, David Wallace

**Affiliations:** Ross Tilley Burn Centre, Sunnybrook Health Sciences Centre, D718, 2075 Bayview Avenue, Toronto M4N 3M5, Canada; Division of Plastic, Reconstructive and Aesthetic Surgery, Department of Surgery, University of Toronto, 149 College Street, Toronto M5T 1P5, Canada; Division of Plastic, Reconstructive and Aesthetic Surgery, Department of Surgery, University of Toronto, 149 College Street, Toronto M5T 1P5, Canada; Ross Tilley Burn Centre, Sunnybrook Health Sciences Centre, D718, 2075 Bayview Avenue, Toronto M4N 3M5, Canada; Division of Plastic, Reconstructive and Aesthetic Surgery, Department of Surgery, University of Toronto, 149 College Street, Toronto M5T 1P5, Canada

**Keywords:** Z-plasty, burn contracture, scar release, hand burn

## Abstract

First webspace contractures are common indications for reconstructive burn surgery. Commonly performed procedures for this indication include either the four- or five-flap variations of the z-plasty, which involves the transposition of flaps about a central limb in order to obtain greater length, and thus, improve the thumb’s important contribution to coordinated and precise hand function. This paper outlines the predominant reason for favouring the five-flap variation for this indication. This conclusion is derived from the notion that although the gain in length may be greater for the four flap, to make the comparison it assumes that the length of the central limbs are the same for the two techniques. For most cases, however, the central limb cannot exceed much >3 cm for a four-flap z-plasty, while 4 cm may be utilized for a ‘jumping man’ procedure.

## Introduction

Thermal injuries to the hand comprise a sizeable proportion of operative cases admitted to burn centres [1]. Therefore, it is not surprising that release of scar contractures involving the first webspace of the hand are one of the most frequently indicated reconstructive procedure [1–3]. Typically the first webspace forms a distinct border between the thinner grafted dorsal skin and the palm’s thicker and usually un-grafted skin. Various techniques have been applied to address these contractures, and most effective and elegant of these have been z-plasties and their variations. The transposition of flaps using adjacent laxity can lengthen scars and efface webs, especially when the contracted scar is narrow and linear rather than a broad area of unstable scar [3–8].

**Figure 1:**
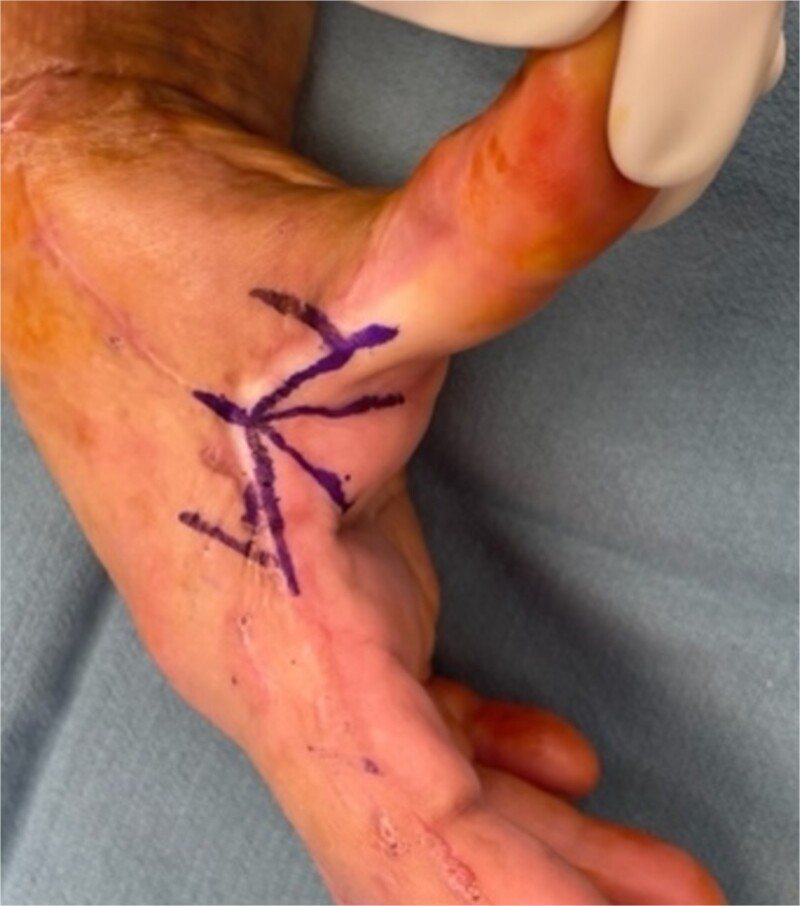
Marking of a five flap Z-plasty along a scar of 3 cm. The V to Y advancement flap originates from the more pliable palmar side.

**Figure 2:**
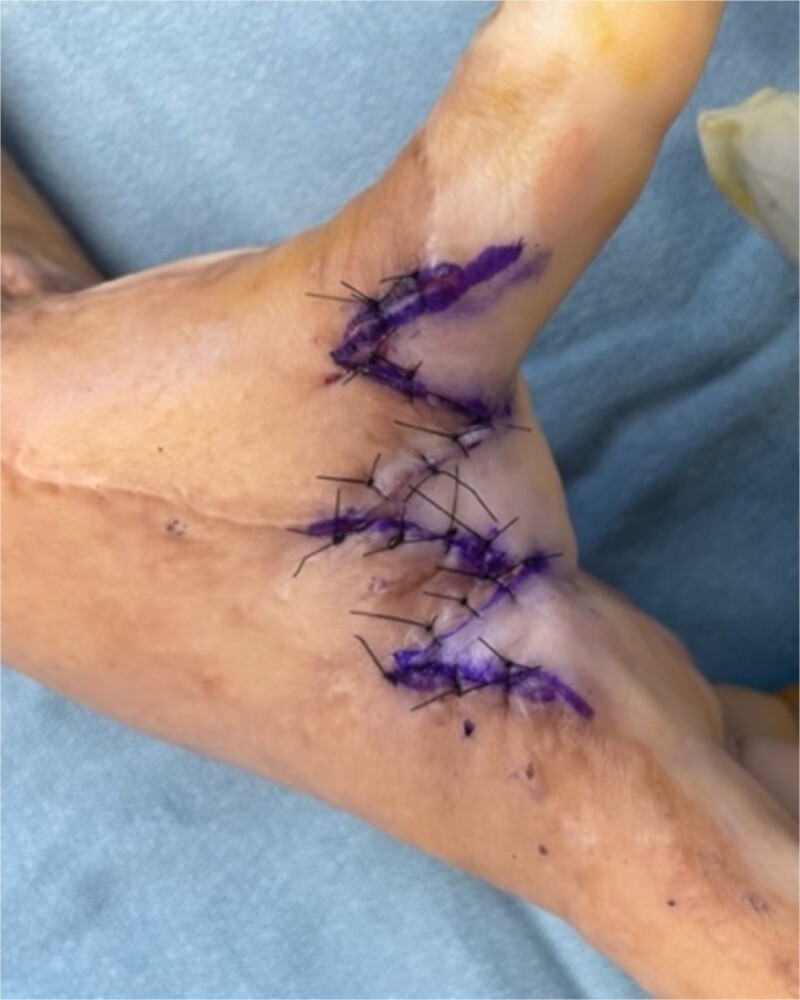
Inset of the five flap Z-plasty in Fig. 1, resulting in increase in length from 3 cm to almost 7 cm.

**Figure 3:**
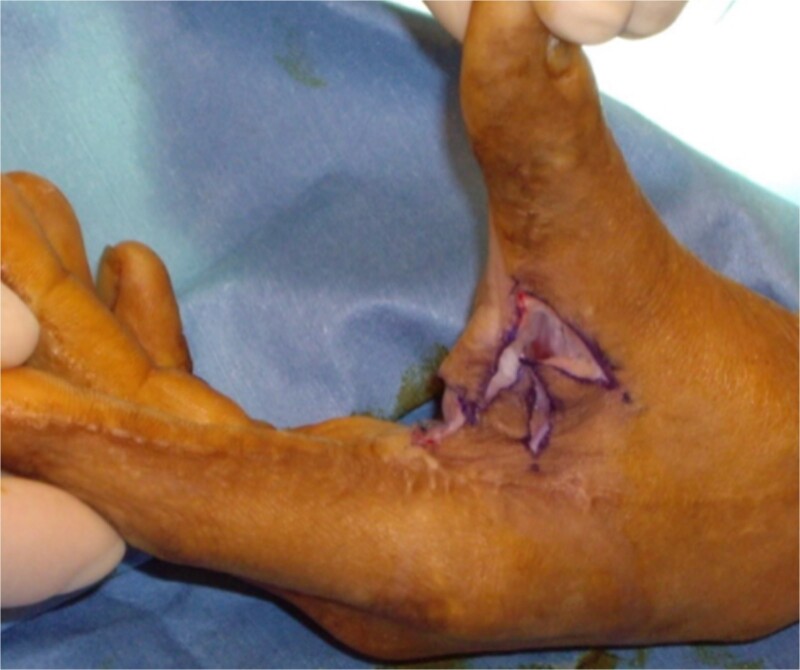
Four flap z-plasty with flaps elevated, prior to suturing. The limbs here are 2 cm, and in this case, the four flaps are designed at 60-degree angles.

**Figure 4:**
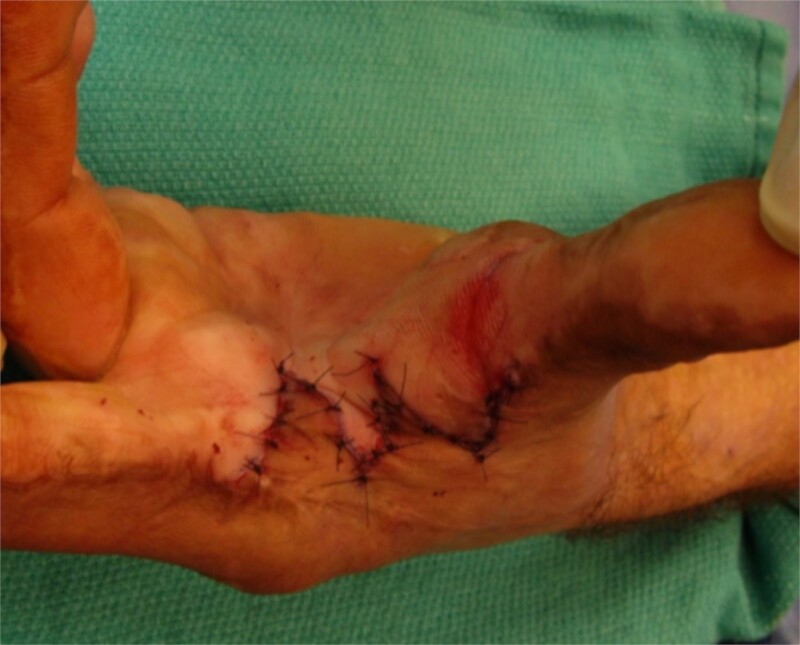
Inset of four flap Z-plasty in Fig. 3. The resulting scar is just over 4 cm in length.

## Case report

All cases of surgical correction by the senior author (AR) of cicatricial first webspace contractures after surgery for burn injury were included in this retrosepctive study over 5 years (1 January 2019 to 31 December 2023). Forty-Five first webspace contractures (after autografting of the dorsum of the hand) in 32 patients were included. Other indications for first webspace contracture, including electrical burns, autografting of the palm, or deepening of the webspace after partial thumb amputations (n = 7) were excluded from consideration, as were cases where the first webpace was released and grafted (n = 4).

The five flap z-plasty (‘jumping man’) flap was used for the majority (n = 36) (Figs 1 and 2), while ~20% (n = 9) were released using the four flap z-plasty (Figs 3 and 4). All central limbs were measured at the start of the surgery, as was the resulting increase in length. The mean central limb utilized for the four-flap z-plasty (120-degree variation) was 3.1 cm, while the mean central limb of a five-flap z-plasty was 4 cm. The mean final length of the contractures was 5.9 and 7.1 cm respectively.

## Discussion

A number of studies describe the theoretical increase in length obtained by various scar contracture releases [5–8]. The 120-degree four flap z-plasty is equivalent to two 60-degree z-plasties in parallel, resulting in 150% additional length (2 × 75% = 150%). The 90-degree variation (four 45-degree flaps) results in a theoretical gain of 120%. Most avoid the latter owing to the very narrow resulting flaps once elevated, especially in skin-grafted areas.

**Figure 5:**
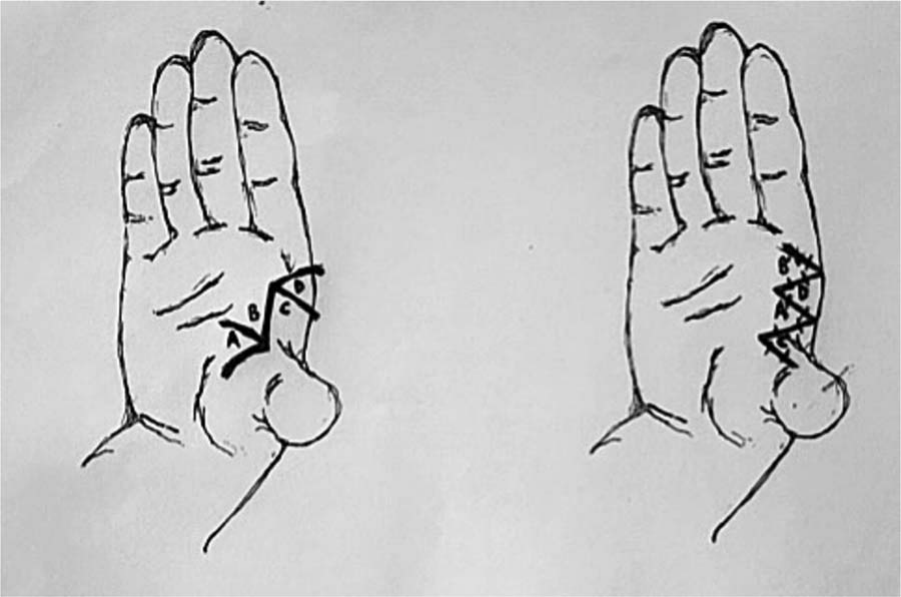
Incisions for webspace release with 60-degree four-flap z-plasty showing initial and final positions of flaps A–D. Note all limbs are of equal length, including the central limb.

**Figure 6:**
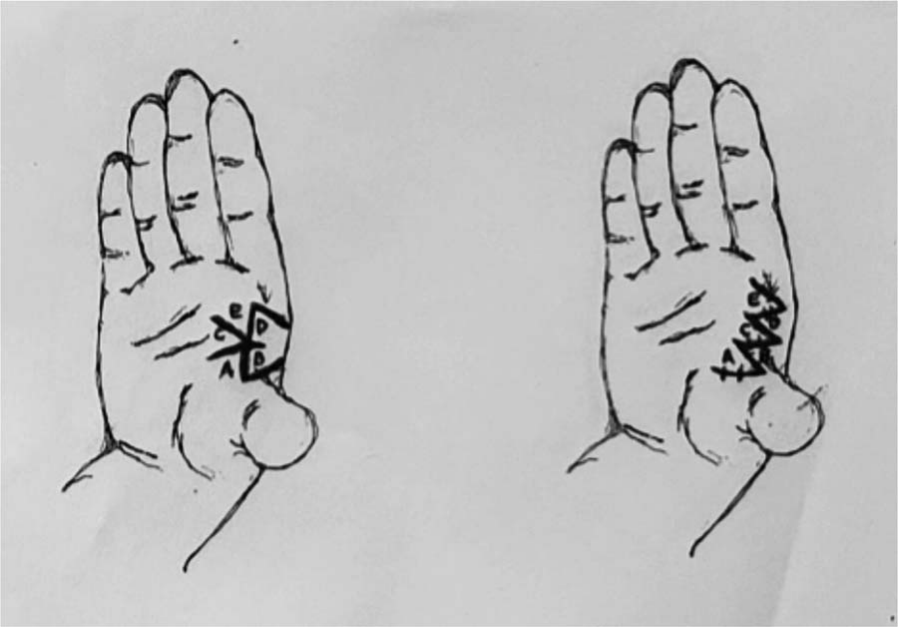
Incisions for webspace release with five-flap z-plasty showing initial and final positions of flaps A–E. Note all limbs are of equal length, except, the central limb (scar contracture), which is double the others. Note the addition of flap C, which is a y-to-v advancement flap.

In comparison, the five-flap z-plasty has two double-opposing 60-degree z-plasties, with the addition of a Y-to-V advancement flap, results in 75% increase for the 60-degree z-plasties and ~50% for the advancement, resulting in a total increase of 125%. Table 1 compares the various types of z-plasties commonly used in the release of webspaces, with increases in length described as a percentage of the central limb, as well as the theoretical new central limb length (assuming the orginal limbs were 1 cm). Figures 5 and 6 show pre and postoperative line drawings of the two techniques, the four-flap and five-flap variations respectively.

**Table 1:** Comparison of the types of Z-plasties, the increase in length of the central limb as a percentage, and the theoretical new length of the central limb (assuming original limbs were 1 cm)

Type of Z-plasty	Increase in length of central limb (%)	New length of central limb (cm)
Simple 60-degree Z-plasty	75	1.75
Four flap Z-plasty with 60-degree angles	150	2.5
Four flap Z-plasty with 45-degree angles	120	2.2
Five flap Z-plasty	125	2.25

Although the four-flap z-plasty may appear to be an attractive option over the five-flap z-plasty, in practice one can seldom address a scar longer than 3 cm because the limbs would also need to be 3 cm, and would invade the hand well beyond the webspace. The five-flap z-plasty allows us to address a longer common limb scar because the flap limbs are shorter. For example, even a 4 cm scar can be released because the limbs are 2 cm each, resulting in a theoretical gain in length of 5 cm (9 cm total).

In conclusion, although both techniques are effective, approaches to lengthen and efface first webspace contractures of the hand, the five-flap z-plasty offers more efficiency and versatility.
